# (*E*)-{[But-2-ene-1,4-diylbis(­oxy)]bis­(4,1-phenyl­ene)}bis­(phenyl­methanone)

**DOI:** 10.1107/S1600536812033624

**Published:** 2012-08-04

**Authors:** Sema Öztürk Yildirim, Ray J. Butcher, Yavuz Köysal, Emrah Birinci

**Affiliations:** a Department of Chemistry, Howard University, 525 College Street NW, Washington, DC 20059, USA; b Department of Physics, Faculty of Sciences, Erciyes University, 38039 Kayseri, Turkey; c Yeşilyurt Demir Çelik Vocational School, Ondokuz Mayis University, Samsun, Turkey; d Department of Chemistry, Karadeniz Technical University, 61080 Trabzon, Turkey

## Abstract

The title mol­ecule, C_30_H_24_O_4_, lies about an inversion center located at the mid-point of the central C=C bond. The diphenyl­methanone unit adopts an all-*trans* conformation. The dihedral angle between the adjacent rings is 53.57 (4)°.

## Related literature

For sterically hindered phenols and secondary aromatic amines as anti­oxidants, see: Rabek (1990 ▶); Pospisil *et al.* (2003 ▶); Wolf & Kaul, (1992 ▶). For synthetic phenolic anti­oxidants, such as butyl­ated hy­droxy­toluene (BHT), butyl­ated hy­droxy­anisole (BHA) or butyl­ated hy­droxy­quinone (TBHQ) as anti­oxidants, see: Omura (1995 ▶). For the ability of phenols to stop the propagation chain during the oxidation process, see: Kumar & Naik (2010 ▶); Findik *et al.* (2011 ▶). For a description of the Cambridge Structural Database, see: Allen (2002 ▶). For the synthesis of the title compound, see: Er *et al.* (2009 ▶).
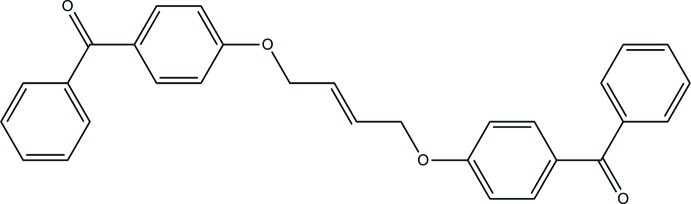


## Experimental

### 

#### Crystal data

C_30_H_24_O_4_
*M* *_r_* = 448.49
Monoclinic, 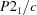

*a* = 24.913 (1) Å
*b* = 7.2586 (3) Å
*c* = 6.1359 (2) Å
β = 95.012 (4)°
*V* = 1105.33 (7) Å^3^
*Z* = 2
Mo *K*α radiation
μ = 0.09 mm^−1^
*T* = 123 K
0.44 × 0.37 × 0.22 mm

#### Data collection

Agilent Xcalibur Ruby Gemini diffractometer
Absorption correction: analytical [*CrysAlis PRO* (Agilent, 2012 ▶), based on expressions derived by Clark & Reid (1995 ▶)] *T*_min_ = 0.976, *T*_max_ = 0.989
8112 measured reflections
2409 independent reflections
1964 reflections with *I* > 2σ(*I*)
*R*_int_ = 0.045

#### Refinement

*R*[*F* ^2^ > 2σ(*F* ^2^)] = 0.053
*wR*(*F* ^2^) = 0.129
*S* = 1.09
2409 reflections
154 parameters
H-atom parameters constrained
Δρ_max_ = 0.25 e Å^−3^
Δρ_min_ = −0.27 e Å^−3^

### 

Data collection: *CrysAlis PRO* (Agilent, 2012 ▶); cell refinement: *CrysAlis PRO*; data reduction: *CrysAlis PRO*; program(s) used to solve structure: *SHELXS97* (Sheldrick, 2008 ▶); program(s) used to refine structure: *SHELXL97* (Sheldrick, 2008 ▶); molecular graphics: *SHELXTL* (Sheldrick, 2008 ▶); software used to prepare material for publication: *SHELXTL*.

## Supplementary Material

Crystal structure: contains datablock(s) I, global. DOI: 10.1107/S1600536812033624/ds2209sup1.cif

Structure factors: contains datablock(s) I. DOI: 10.1107/S1600536812033624/ds2209Isup2.hkl

Supplementary material file. DOI: 10.1107/S1600536812033624/ds2209Isup3.cml

Additional supplementary materials:  crystallographic information; 3D view; checkCIF report

## Figures and Tables

**Fig. 1.**
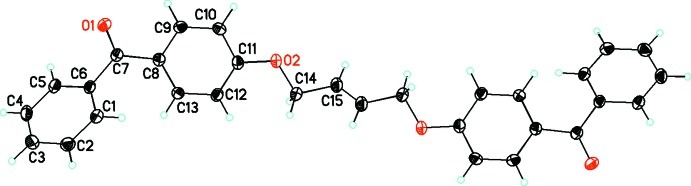
A view of the molecular structure of the title molecule, with the atom-numbering. Displacement ellipsoids are drawn at the 50% probability level [symmetry code: (A) = 2 - x, -y, -z].

**Fig. 2.**
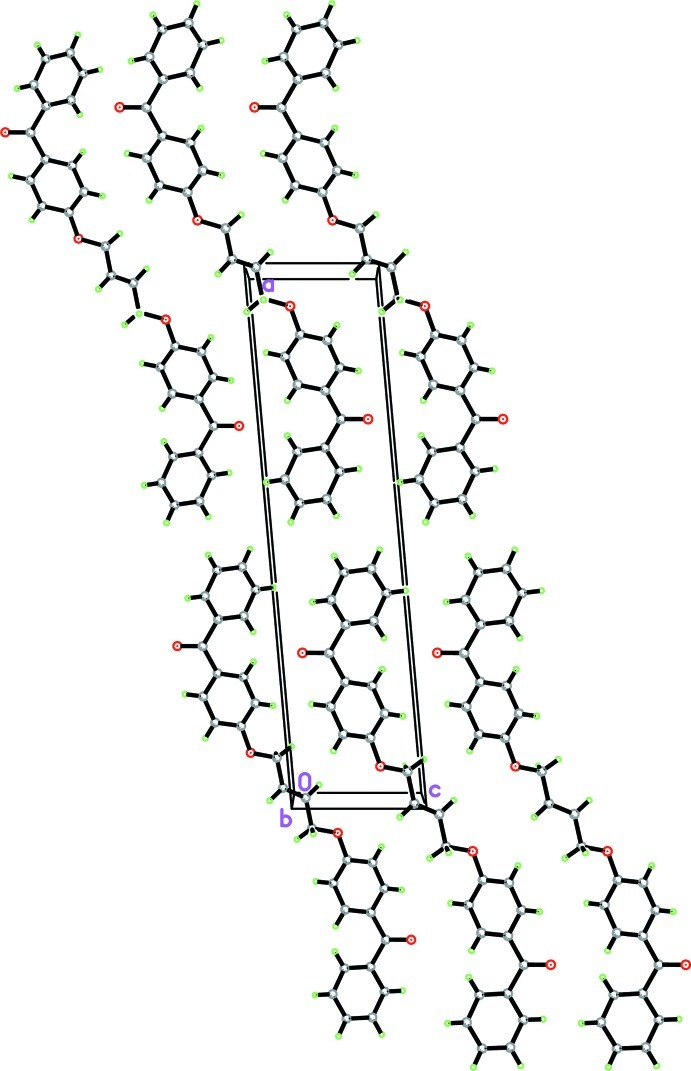
Packing diagram of the title compound with along the b axis.

## supplementary crystallographic information

### Comment

Antioxidants are chemical compounds that can quench reactive radical intermediates formed during oxidative reactions. The primary antioxidants essentially comprise of sterically hindered phenols and secondary aromatic amines (Rabek, 1990; Pospisil *et al.*, 2003; Wolf & Kaul, 1992). Phenols have been utilized extensively for food preservation. Synthetic phenolic antioxidants, such as butylated hydroxytoluene (BHT), butylated hydroxyanisole (BHA) or butylated hydroxyquinone (TBHQ) possess good antioxidant capacity (Omura, 1995). The main structural feature responsible for the antioxidative and free radical scavenging activity of phenolic derivatives is the phenolic hydroxyl group. Phenols are able to donate the hydrogen atom of the phenolic OH to the free radicals, thus stopping the propagation chain during the oxidation process (Kumar & Naik, 2010; Findik, *et al.*, 2011).

In view of the importance of phenolate compounds as antioxidants the structure of (*E*)-((but-2-ene-1,4-diylbis(oxy))bis(4,1-phenylene))bis(phenylmethanone) was determined. This molecule, C_30_H_24_O_4_, (Fig. 1), lies on an inversion centre, which passes through middle point of the C15=C15A double bond of the aliphatic chain, giving one half-molecule per asymmetric unit. As a consequence of this symmetry, the diphenylmethanone adopts an all-*trans* conformation. The molecular structure is not planar. The O2 C14 C15 C15A (C15A generated by 2 - *x*, -*y*, -*z*) torsion angle is 123.3 (3) ° and the dihedral angle between the planes of the aromatic rings (C1/C6 to C8/C13) is 53.57 (4) ° [for the non-H atoms, maximum deviation = -0.015 (1) Å for C10]. Bond lengths and angles can be regarded as normal (Allen, 2002). There are no significant C–H···O contacts.

### Experimental

Title compound was synthesized by published methods (Er *et al.*, 2009). Crystals were grown by slow evaporation of an ethanol/acetone mixed solution.

### Refinement

All H-atoms were positioned geometrically with C—H = 0.93 or 0.97 Å and refined using a riding model with *U*_iso_(H) = 1.2*U*_eq_(C).

### Crystal data

**Table:**

C_30_H_24_O_4_	*F*(000) = 472
*M**_r_* = 448.49	*D*_x_ = 1.348 Mg m^−^^3^
Monoclinic, *P*2_1_/*c*	Mo *K*α radiation, λ = 0.71073 Å
Hall symbol: -P 2ybc	Cell parameters from 3302 reflections
*a* = 24.913 (1) Å	θ = 3.3–28.6°
*b* = 7.2586 (3) Å	µ = 0.09 mm^−^^1^
*c* = 6.1359 (2) Å	*T* = 123 K
β = 95.012 (4)°	Pyrimidal, colorless
*V* = 1105.33 (7) Å^3^	0.44 × 0.37 × 0.22 mm
*Z* = 2	

### Data collection

**Table:**

Agilent Xcalibur Ruby Gemini diffractometer	2409 independent reflections
Radiation source: fine-focus sealed tube	1964 reflections with *I* > 2σ(*I*)
Graphite monochromator	*R*_int_ = 0.045
Detector resolution: 10.5081 pixels mm^-1^	θ_max_ = 28.6°, θ_min_ = 3.3°
ω scans	*h* = −27→31
Absorption correction: analytical [*CrysAlis PRO* (Agilent, 2012), based on expressions derived by Clark & Reid (1995)]	*k* = −9→7
*T*_min_ = 0.976, *T*_max_ = 0.989	*l* = −8→7
8112 measured reflections	

### Refinement

**Table:**

Refinement on *F*^2^	Primary atom site location: structure-invariant direct methods
Least-squares matrix: full	Secondary atom site location: difference Fourier map
*R*[*F*^2^ > 2σ(*F*^2^)] = 0.053	Hydrogen site location: inferred from neighbouring sites
*wR*(*F*^2^) = 0.129	H-atom parameters constrained
*S* = 1.09	*w* = 1/[σ^2^(*F*_o_^2^) + (0.0494*P*)^2^ + 0.5741*P*] where *P* = (*F*_o_^2^ + 2*F*_c_^2^)/3
2409 reflections	(Δ/σ)_max_ = 0.001
154 parameters	Δρ_max_ = 0.25 e Å^−^^3^
0 restraints	Δρ_min_ = −0.27 e Å^−^^3^

### Special details

**Table:**

Experimental. Absorption correction: analytical numeric absorption correction using a multifaceted crystal model based on expressions derived by Clark & Reid (1995).
Geometry. All e.s.d.'s (except the e.s.d. in the dihedral angle between two l.s. planes) are estimated using the full covariance matrix. The cell e.s.d.'s are taken into account individually in the estimation of e.s.d.'s in distances, angles and torsion angles; correlations between e.s.d.'s in cell parameters are only used when they are defined by crystal symmetry. An approximate (isotropic) treatment of cell e.s.d.'s is used for estimating e.s.d.'s involving l.s. planes.
Refinement. Refinement of *F*^2^ against ALL reflections. The weighted *R*-factor *wR* and goodness of fit *S* are based on *F*^2^, conventional *R*-factors *R* are based on *F*, with *F* set to zero for negative *F*^2^. The threshold expression of *F*^2^ > σ(*F*^2^) is used only for calculating *R*-factors(gt) *etc*. and is not relevant to the choice of reflections for refinement. *R*-factors based on *F*^2^ are statistically about twice as large as those based on *F*, and *R*- factors based on ALL data will be even larger.

### Fractional atomic coordinates and isotropic or equivalent isotropic displacement parameters (Å^2^)

**Table:**

	*x*	*y*	*z*	*U*_iso_*/*U*_eq_	
O1	0.71419 (5)	0.00150 (18)	0.81909 (19)	0.0279 (3)	
O2	0.92164 (4)	−0.01342 (16)	0.31446 (19)	0.0240 (3)	
C1	0.65514 (7)	−0.0714 (2)	0.2731 (3)	0.0214 (4)	
H1A	0.6853	−0.1202	0.2080	0.026*	
C2	0.60418 (7)	−0.0791 (2)	0.1610 (3)	0.0243 (4)	
H2A	0.5994	−0.1345	0.0203	0.029*	
C3	0.56033 (7)	−0.0055 (2)	0.2551 (3)	0.0264 (4)	
H3A	0.5254	−0.0123	0.1796	0.032*	
C4	0.56713 (7)	0.0778 (2)	0.4592 (3)	0.0255 (4)	
H4A	0.5371	0.1313	0.5209	0.031*	
C5	0.61760 (6)	0.0832 (2)	0.5730 (3)	0.0222 (4)	
H5A	0.6221	0.1381	0.7139	0.027*	
C6	0.66211 (6)	0.0077 (2)	0.4805 (3)	0.0193 (4)	
C7	0.71442 (6)	0.0017 (2)	0.6198 (3)	0.0196 (4)	
C8	0.76709 (6)	−0.0056 (2)	0.5208 (3)	0.0189 (4)	
C9	0.81067 (6)	−0.0885 (2)	0.6439 (3)	0.0203 (4)	
H9A	0.8053	−0.1436	0.7808	0.024*	
C10	0.86106 (6)	−0.0910 (2)	0.5686 (3)	0.0207 (4)	
H10A	0.8900	−0.1516	0.6505	0.025*	
C11	0.86966 (6)	−0.0043 (2)	0.3717 (3)	0.0195 (4)	
C12	0.82714 (6)	0.0799 (2)	0.2468 (3)	0.0207 (4)	
H12A	0.8330	0.1391	0.1128	0.025*	
C13	0.77587 (6)	0.0758 (2)	0.3220 (3)	0.0195 (4)	
H13A	0.7465	0.1297	0.2357	0.023*	
C14	0.93354 (7)	0.0764 (3)	0.1161 (3)	0.0251 (4)	
H14A	0.9271	0.2106	0.1262	0.030*	
H14B	0.9103	0.0271	−0.0097	0.030*	
C15	0.99148 (7)	0.0394 (3)	0.0874 (3)	0.0265 (4)	
H15A	1.0176	0.0748	0.2019	0.032*	

### Atomic displacement parameters (Å^2^)

**Table:**

	*U*^11^	*U*^22^	*U*^33^	*U*^12^	*U*^13^	*U*^23^
O1	0.0252 (7)	0.0416 (8)	0.0173 (6)	−0.0015 (5)	0.0036 (5)	−0.0004 (5)
O2	0.0154 (6)	0.0328 (7)	0.0242 (6)	0.0014 (5)	0.0045 (5)	0.0017 (5)
C1	0.0218 (8)	0.0217 (8)	0.0215 (8)	−0.0006 (6)	0.0055 (7)	0.0005 (7)
C2	0.0264 (9)	0.0263 (9)	0.0201 (8)	−0.0031 (7)	0.0013 (7)	0.0000 (7)
C3	0.0207 (8)	0.0305 (10)	0.0276 (9)	−0.0018 (7)	−0.0002 (7)	0.0048 (7)
C4	0.0185 (8)	0.0290 (9)	0.0301 (9)	−0.0005 (7)	0.0076 (7)	0.0020 (7)
C5	0.0214 (8)	0.0248 (9)	0.0211 (8)	−0.0021 (7)	0.0064 (7)	−0.0010 (7)
C6	0.0179 (8)	0.0207 (8)	0.0198 (8)	−0.0017 (6)	0.0050 (6)	0.0019 (6)
C7	0.0199 (8)	0.0198 (8)	0.0195 (8)	−0.0017 (6)	0.0036 (6)	−0.0007 (6)
C8	0.0191 (8)	0.0188 (8)	0.0191 (8)	−0.0011 (6)	0.0024 (6)	−0.0023 (6)
C9	0.0231 (8)	0.0216 (8)	0.0162 (8)	−0.0024 (6)	0.0009 (6)	−0.0003 (6)
C10	0.0199 (8)	0.0215 (9)	0.0201 (8)	0.0004 (6)	−0.0021 (6)	−0.0008 (6)
C11	0.0167 (8)	0.0212 (8)	0.0208 (8)	−0.0015 (6)	0.0025 (6)	−0.0053 (6)
C12	0.0205 (8)	0.0220 (8)	0.0199 (8)	−0.0008 (6)	0.0045 (6)	0.0013 (6)
C13	0.0187 (8)	0.0212 (8)	0.0185 (8)	0.0013 (6)	0.0012 (6)	−0.0001 (6)
C14	0.0204 (8)	0.0331 (10)	0.0225 (9)	0.0002 (7)	0.0058 (7)	0.0010 (7)
C15	0.0174 (8)	0.0357 (10)	0.0267 (9)	−0.0029 (7)	0.0039 (7)	−0.0015 (7)

### Geometric parameters (Å, º)

**Table:**

O1—C7	1.223 (2)	C8—C13	1.390 (2)
O2—C11	1.3726 (19)	C8—C9	1.403 (2)
O2—C14	1.435 (2)	C9—C10	1.376 (2)
C1—C2	1.391 (2)	C9—H9A	0.9500
C1—C6	1.393 (2)	C10—C11	1.395 (2)
C1—H1A	0.9500	C10—H10A	0.9500
C2—C3	1.387 (3)	C11—C12	1.393 (2)
C2—H2A	0.9500	C12—C13	1.395 (2)
C3—C4	1.388 (3)	C12—H12A	0.9500
C3—H3A	0.9500	C13—H13A	0.9500
C4—C5	1.384 (2)	C14—C15	1.494 (2)
C4—H4A	0.9500	C14—H14A	0.9900
C5—C6	1.401 (2)	C14—H14B	0.9900
C5—H5A	0.9500	C15—C15^i^	1.318 (4)
C6—C7	1.496 (2)	C15—H15A	0.9500
C7—C8	1.494 (2)		
			
C11—O2—C14	117.70 (12)	C10—C9—C8	120.69 (15)
C2—C1—C6	120.26 (16)	C10—C9—H9A	119.7
C2—C1—H1A	119.9	C8—C9—H9A	119.7
C6—C1—H1A	119.9	C9—C10—C11	119.94 (14)
C3—C2—C1	119.77 (16)	C9—C10—H10A	120.0
C3—C2—H2A	120.1	C11—C10—H10A	120.0
C1—C2—H2A	120.1	O2—C11—C12	124.69 (15)
C2—C3—C4	120.35 (16)	O2—C11—C10	114.82 (14)
C2—C3—H3A	119.8	C12—C11—C10	120.48 (15)
C4—C3—H3A	119.8	C11—C12—C13	118.87 (15)
C5—C4—C3	120.08 (16)	C11—C12—H12A	120.6
C5—C4—H4A	120.0	C13—C12—H12A	120.6
C3—C4—H4A	120.0	C8—C13—C12	121.18 (15)
C4—C5—C6	120.06 (16)	C8—C13—H13A	119.4
C4—C5—H5A	120.0	C12—C13—H13A	119.4
C6—C5—H5A	120.0	O2—C14—C15	106.96 (13)
C1—C6—C5	119.44 (15)	O2—C14—H14A	110.3
C1—C6—C7	122.90 (15)	C15—C14—H14A	110.3
C5—C6—C7	117.43 (15)	O2—C14—H14B	110.3
O1—C7—C8	119.17 (14)	C15—C14—H14B	110.3
O1—C7—C6	119.44 (15)	H14A—C14—H14B	108.6
C8—C7—C6	121.38 (14)	C15^i^—C15—C14	123.9 (2)
C13—C8—C9	118.77 (15)	C15^i^—C15—H15A	118.1
C13—C8—C7	123.58 (14)	C14—C15—H15A	118.1
C9—C8—C7	117.50 (14)		
			
C6—C1—C2—C3	0.8 (3)	C6—C7—C8—C9	−152.96 (15)
C1—C2—C3—C4	0.9 (3)	C13—C8—C9—C10	−0.7 (2)
C2—C3—C4—C5	−1.9 (3)	C7—C8—C9—C10	−176.32 (15)
C3—C4—C5—C6	1.3 (3)	C8—C9—C10—C11	2.4 (2)
C2—C1—C6—C5	−1.5 (2)	C14—O2—C11—C12	2.6 (2)
C2—C1—C6—C7	173.00 (15)	C14—O2—C11—C10	−178.67 (14)
C4—C5—C6—C1	0.4 (2)	C9—C10—C11—O2	179.11 (14)
C4—C5—C6—C7	−174.34 (15)	C9—C10—C11—C12	−2.1 (2)
C1—C6—C7—O1	−150.21 (16)	O2—C11—C12—C13	178.62 (15)
C5—C6—C7—O1	24.4 (2)	C10—C11—C12—C13	−0.1 (2)
C1—C6—C7—C8	29.3 (2)	C9—C8—C13—C12	−1.5 (2)
C5—C6—C7—C8	−156.15 (15)	C7—C8—C13—C12	173.86 (15)
O1—C7—C8—C13	−148.92 (17)	C11—C12—C13—C8	1.9 (2)
C6—C7—C8—C13	31.6 (2)	C11—O2—C14—C15	−177.68 (14)
O1—C7—C8—C9	26.5 (2)	O2—C14—C15—C15^i^	123.3 (2)

Symmetry code: (i) −*x*+2, −*y*, −*z*.
